# The impact of host plant species on instar duration and body weight of nymphal *Lycorma delicatula*

**DOI:** 10.3389/finsc.2022.1110480

**Published:** 2023-01-19

**Authors:** Devin Kreitman, Melody A. Keena, Anne L. Nielsen, George Hamilton

**Affiliations:** ^1^ Entomology Department, Rutgers, The State University of New Jersey, Brunswick, NJ, United States; ^2^ Northern Research Station, USDA Forest Service, Hamden, CT, United States

**Keywords:** phenology, survival, development, hostplant, lanternfly

## Abstract

The spotted lanternfly, *Lycorma delicatula* (White) (Hemiptera: Fulgoridae), is an invasive species of planthopper that was introduced to North America and is a threat to multiple industries. Nymphs and egg masses were collected to assess each instar’s rate of development at a constant temperature of 25°C on the following hosts: *Ailanthus altissima* (Miller) (Sapindales: Simaroubaceae), *Vitis labrusca* (L.) (Vitales: Vitaceae), *Salix babylonica* (L.) (Malpighiales: Salicaceae), *Acer rubrum* (L.) (Sapindales: Sapindaceae), *Celastrus orbiculata* (Thunberg) (Celastrales: Celastraceae), *Ocimum basilicum* (L.) (Lamiales: Lamiaceae), and *Rosa multiflora* (Thunberg) (Rosales: Rosaceae). Host plant species was found to have a significant effect on developmental time for nymphs in the first through third instars, as well as on nymphal survival. Nymphs failed to develop through the second instar on *O. basilicum* and the third and fourth instars on *A. rubrum*. Host plant species also had a significant effect on the mean weight of nymphs in the first, second, and fourth instars (but not in the third instar), and on the hind tibia length and forewing width of adult nymphs. This variability in *L. delicatula* developmental time by host plant species can potentially impact phenology models, which should be updated to reflect these new insights. Rearing practices should also be refined to account for host plant influences on the physiology of *L. delicatula*.

## Introduction

The spotted lanternfly, *Lycorma delicatula* (White) (Hemiptera: Fulgoridae), is an invasive species of planthopper that was first detected in the United States in the summer of 2014. *L. delicatula* is native to China, Vietnam, and India; the United States is one of three countries invaded by this species, together with Japan and South Korea (1). Since its initial detection in Pennsylvania, *L. delicatula* has spread across the northeastern region of the United States and is now established in multiple states (1).

*L. delicatula* has four instars. The first-instar nymphs start to emerge from eggs in late April in North America (2). Nymphs of the first three instars are black and white, and fourth-instar nymphs are black, white, and red in color. Adults appear around mid-July and lay eggs from early September until temperatures are low enough to kill them. The eggs are deposited in grayish to tan-colored egg masses on various substrates, such as bark, stone, wood fences, and brick, on which the egg masses overwinter until the following spring.

*L. delicatula* has a broad host range consisting of 103 plant species (2). Despite this, *L. delicatula* has a preferred host, which is the tree of heaven, *Ailanthus altissima* (Miller) (Sapindales: Simarobaceae) (3). Recently, it was found that *L. delicatula* does not require *A. altissima* to complete its lifecycle, but that the removal of *A. altissima* from its diet is associated with reduced fitness (4). However, despite being widespread and commonly found in disturbed sites, *A. altissima* is not always available as a host for *L. delicatula*.

External temperature has a major influence on the development and growth of insects; however, other factors can also influence their growth. Previous research has shown that the host plant can affect an insect’s phenology and should be considered in phenology models (5, 6). For example, the larvae of the Oriental fruit moth, *Grapholita molesta* (Busck) (Lepidoptera: Tortricidae), develop more quickly when feeding on *Prunus persica* (L.) (Rosales: Rosaceae) than when feeding on *Malus domestica* (Borkhausen) (Rosales: Rosaceae) (7). Likewise, nymphs of the brown marmorated stink bug, *Halyomorpha halys* (Stål) (Hemiptera: Pentatomidae), from the second instar onward were found to develop more quickly when reared on *P. persica* than when reared on *M. domestica* (8).

The phenology of *L. delicatula* has been previously determined on *A. altissima* and *Parthenocissus quinquefolia* (L.) (Vitales: Vitaceae) (9, 10). In the latter study, when *L. delicatula* was reared on *P. quinquefolia* at room temperature (assumed to be slightly above 20°C), it was found that the developmental duration of the first-, second-, third-, and fourth-instar nymphs was 18.8, 20.9, 20.8, and 22.2 days, respectively (10). When *L. delicatula* was reared on *A. altissima* at that temperature, it was found that the duration of the first-, second-, and third-instar nymphs was 23.4, 24.0, and 40.4 days, respectively (9). The data for the fourth-instar nymphs were separated by sex, with male and female nymphs completing that instar within 39.5 and 50.1 days, respectively. These differences in results, with *L. delicatula* taking less time to develop on *P. quinquefolia* than on *A. altissima* at 20°C, suggest that host plant species also influences their development. In addition, fourth-instar nymphs were found to take fewer days to develop at 25°C when they were reared on fox grape, *Vitis labrusca* (L.) (Vitales: Vitaceae), than on *A. altissima* in an unpublished study (8), a finding which further stresses the importance of determining the developmental rate of *L. delicatula* on different host plants.

To further understand the effect of host plant species on the development of *L. delicatula*, it is important to rear nymphs on a variety of different host plants. In this study, the survival and development of nymphs and the weight and size of *L. delicatula* adult insects were examined using one of the following plants as a host: tree of heaven (*A. altissima*), fox grape (*V. labrusca*), weeping willow [*Salix babylonica* (L.) (Malpighiales: Salicaceae)], red maple [*Acer rubrum* (L.) (Sapindales: Sapindaceae)], Oriental bittersweet [*Celastrus orbiculata* (Thunberg) (Celastrales: Celastraceae)], basil [*Ocimum basilicum* (L.) (Lamiales: Lamiaceae)], and multiflora rose [*Rosa multiflora* (Thunberg) (Rosales: Rosaceae)]. The results from this study will help to further advance phenology models for this insect.

## Methods

### Source populations

On 17 June 2020, *L. delicatula* first-instar (*n* = 140) and second-instar (*n* = 63) nymphs were collected at a site in Hunterdon County, New Jersey, USA (Riegelsville, NJ). The site had *Vitis* spp., *Rosa* spp., *C. orbiculata*, *A. altissima*, *Celtis occidentalis* (L.) (Rosales: Cannabaceae), and *Juglans nigra* (Fagales: Juglandaceae), as well as other assorted unidentified shrubbery. The nymphs were found mostly in the shade, and egg masses were observed on site. The nymphs were transferred to a quarantine facility located in Ansonia, Connecticut, USA, as per the terms of the US Department of Agriculture Animal and Plant Health Inspection Service (USDA APHIS) permits, in containers containing a single 50- to 70-cm-long sprig of wild grape, *Vitis volpina* L. (Vitales: Vitaceae), to sustain them for the trip. At the quarantine facility, the nymphs were sorted by instar and placed into a large mesh cage (60 cm × 60 cm × 120 cm; BugDorm 6S620, MegaView Science Co., Ltd, Taichung, Taiwan) with two or three 100-cm-tall potted *A. altissima* plants and a single *V. labrusca* plant and kept at 25°C with a photoperiod of 16 h : 8 h (L : D) and a relative humidity between 60% and 80%. Once the nymphs began molting to the next instar, 10 that molted on the same day were taken and set up in a smaller 32.5 cm × 32.5 cm × 77.0 cm mesh cage (BugDorm 4S3074, MegaView Science Co., Ltd, Taichung, Taiwan) containing two host plants of the same species for use in experiments. These smaller cages were kept at the same photoperiod, temperature, and humidity as the other larger cages. Any additional nymphs that molted were transferred to the large BugDorm cages and allowed to develop into later instars.

### Plant rearing

The host plants that were used were selected for a variety of reasons. *Ailanthus altissima* was selected because it is the preferred host for *L. delicatula*, making it a good reference for comparison with other host plants. As *L. delicatula* poses a significant threat to wine grapes, it is important to determine if being reared on *V. labrusca* influences its developmental rate. *Salix babylonica* was selected because it is a common landscape tree and was one of the trees used in the study that showed that *L. delicatula* could complete development without *A. altissima* (4). *Celastrus orbiculata* was selected based on previous literature findings indicating that *L. delicatula*, in the early instars, commonly used it as a host. *Ocimum basilicum* is a common garden plant and *R. multiflora* is a common forest plant, and it has been found that *L. delicatula* feeds on both plants.

*A. altissima* was grown from seeds collected in Wallingford, Connecticut, USA, in October 2019. Seeds were initially planted in Jiffy Plugs and then transferred to tree pots measuring 7.6 cm × 7.6 cm × 20.3 cm (CN-SS-TP-308, Greenhouse Megastore, Danville, IL, USA) filled with soil (Premier BK25, Promix M, Premier Horticultural Inc., Quakertown, PA, USA) after sprouting. The *A. altissima* seedlings were provided with 5–10 g (the amount was dependent on the size of the pot) of Osmocote fertilizer (ICL Specialty Fertilizers, Summerville, SC, USA) when they were first put into the tree pots, and monthly thereafter. Otherwise, the *A. altissima* seedlings were reared as described in Kreitman et al. (9).

*Celastrus orbiculate* was grown from cuttings from multiple plants obtained from the towns of Wallingford and Ansonia (CT, USA). *Rosa multiflora* was grown from cuttings obtained from multiple plants from Wallingford, Connecticut, USA, and from at least 10 individual plants from Ansonia, Connecticut, USA. Both Celastrus orbiculate and Rosa multiflora were obtained during the summer of 2020. *Ocimum basilicum* plants were grown from “hybrid herb, basil Prospera organic” seeds purchased from Seedway, LLC (Hall, New York, NY, USA) using the same method as for the *A. altissima* plants. *Celastrus orbiculate*, *R. multiflora*, and *O. basilicum* were all grown in the same soil and tree pots as the *A. altissima* plants.

The *A. rubrum* and *S. babylonica* plants were purchased from Cold Stream Farm LLC (Freesoil, MI, USA) in late March 2020. The *V. labrusca* bare-root plants were purchased from Double A Vineyard (Fredonia, NY, USA) and were shipped in the spring of 2020.

### The effect of different host plant species on the development of nymphal *Lycorma delicatula*

#### Nymphal rearing in 2020

For this first year, the host plants used were *A. altissima*, *V. labrusca*, *S. babylonica*, and *A. rubrum*. Three cages of each host plant treatment were set up, with 10 second-instar nymphs or 10 third-instar nymphs per cage, both sourced from the rearing cages containing the field-collected nymphs. For the fourth-instar nymphs, five nymphs that molted on the same day were placed in a small cage for each host with 10 replicates of each over a period of 12 days. Each cage started with two plants, and new plants of the same host were added to the cages every 7 days for the second- through third-instar nymphs, and every 4 days for the fourth-instar nymphs. Nymphs were monitored daily for survival and molting, which was confirmed by a cast skin. Any newly molted nymphs were removed from the cages, weighed, and then preserved by freezing for later sexing. The second-instar nymphs were preserved in ethanol, and, therefore, we were unable to determine their sex. Measurements of forewing length, forewing width, and hind tibia length were taken for all frozen adult nymphs using a dissecting microscope.

#### Nymphal rearing in 2021

In 2021, we evaluated only the first- and second-instar nymphs of the hosts. The nymphs were hatched (first instars) or reared (second instars) from field-collected egg masses. The egg masses were collected by removing both the egg mass and the bark substrate that it was on using a chisel and hammer, from two sites in Pennsylvania and one site in New Jersey, on 20 October 2020 (**Table 1**). These egg masses were held individually in 60 mm × 15 mm Petri dishes (Falcon 351007, Becton Dickinson Labware, Franklin Lakes, NJ, USA) at 15°C until they hatched. On hatching, 20 nymphs that hatched from egg masses on the same day, from the same collection site, were placed in a small BugDorm cage (32.5 cm × 32.5 cm × 77.0 cm) containing two plants of the same species. The species of plants used were *A. altissima*, *V. labrusca*, *S. babylonica*, *A. rubrum*, *C. orbiculata*, *O. basilicum*, and *R. multiflora*. The range of host plant species was expanded in 2021 because of the promising preliminary results in 2020. Additional hatch from those egg masses was placed in the larger BugDorm cages (60 cm × 60 cm × 120 cm) with two or three 100-cm-tall-potted *A. altissima* plants and a single *V. labrusca* plant, and kept at a temperature of 25°C for rearing to be used as second-instar nymphs. For both the first- and second-instar nymphs, two cages were set up with nymphs from the New Jersey site, and one cage was set up with nymphs from each Pennsylvania site, for a total of four cages for each host. Voucher specimens were preserved in a freezer for reference, in addition to the voucher specimens of adult *L. delicatula* that were deposited at the Entomology Division, Yale Peabody Museum of Natural History, New Haven, Connecticut, USA.

**Table 1:** Approximate locations (latitude and longitude), collection date, and hosts from which the egg masses of *Lycorma delicatula* used in this study were obtained.

Collection location	Collection date	Host (number of egg masses)	Latitude	Longitude
Spruce Run Reservoir, Clinton, NJ, USA	10 October 2020	*Betula pendula* Roth (93) and dead trees (23)	40°39′47.03″N	74°55′36.02″W
The Woodlands, Philadelphia, PA, USA	10 October 2020	*Prunus* spp. (110), *Broussonentia papyrifera* (L. Vent.) (Rosales: Moraceae) (8), *Acer platanoides* (L.) (Sapindales: Sapindaceae) (7), and *Crataegus* spp. (Rosales: Rosaceae) (10)	39°5′45.86″N	75°12′19.37″W
Neshaminy State Park, Bensalem, PA, USA	10 October 2020	*Betula nigra* (L.) (Fagales: Betulaceae) (33), *Betula lenta* (L.) (Fagales: Betulaceae) (18), *Acer rubrum* (25), *Prunus* spp. (18), and *Pinus strobus* (L.) (Pinales: Pinaceae) (24)	40°4′31.87″N	75°55′0.59″W

### Statistical analyses

Statistical analyses were performed using SAS 9.4 (11). Data did not fit assumptions of normality per the Shapiro–Wilk and Anderson–Darling tests. PROC UNIVARIATE was then used to assess the fit of the data to a gamma distribution. Each model was fitted to a gamma distribution with a log-link function because the response variables had long right tails. PROC GLIMMIX was used to evaluate the fixed effect of host on the duration and body mass of each instar. If the sex was known, the fixed effects of sex and the interaction of sex and host plant species were added to the model. The state(s) in which the egg masses were collected, and of the cages, were treated as random effects. PROC GLIMMIX with a beta distribution and logit link function was used to evaluate the effects of host on the overall survival for each instar. Percentage survival was calculated for each cage. Values of 1 were replaced with 0.9999, and values of 0 were replaced with 0.0001, because the beta curve allows only values between 1 and 0. Differences among means were determined using Tukey–Kramer *post hoc* analysis and a α value equal to 0.05. Statistical comparisons of nymphal survival curves between host plants for each instar were carried out using a Peto–Wilcoxon test in Statistix 10.0 (12). For this analysis, any nymphs that molted or were inadvertently killed were censored.

## Results

### Survival

**Figure 1:**
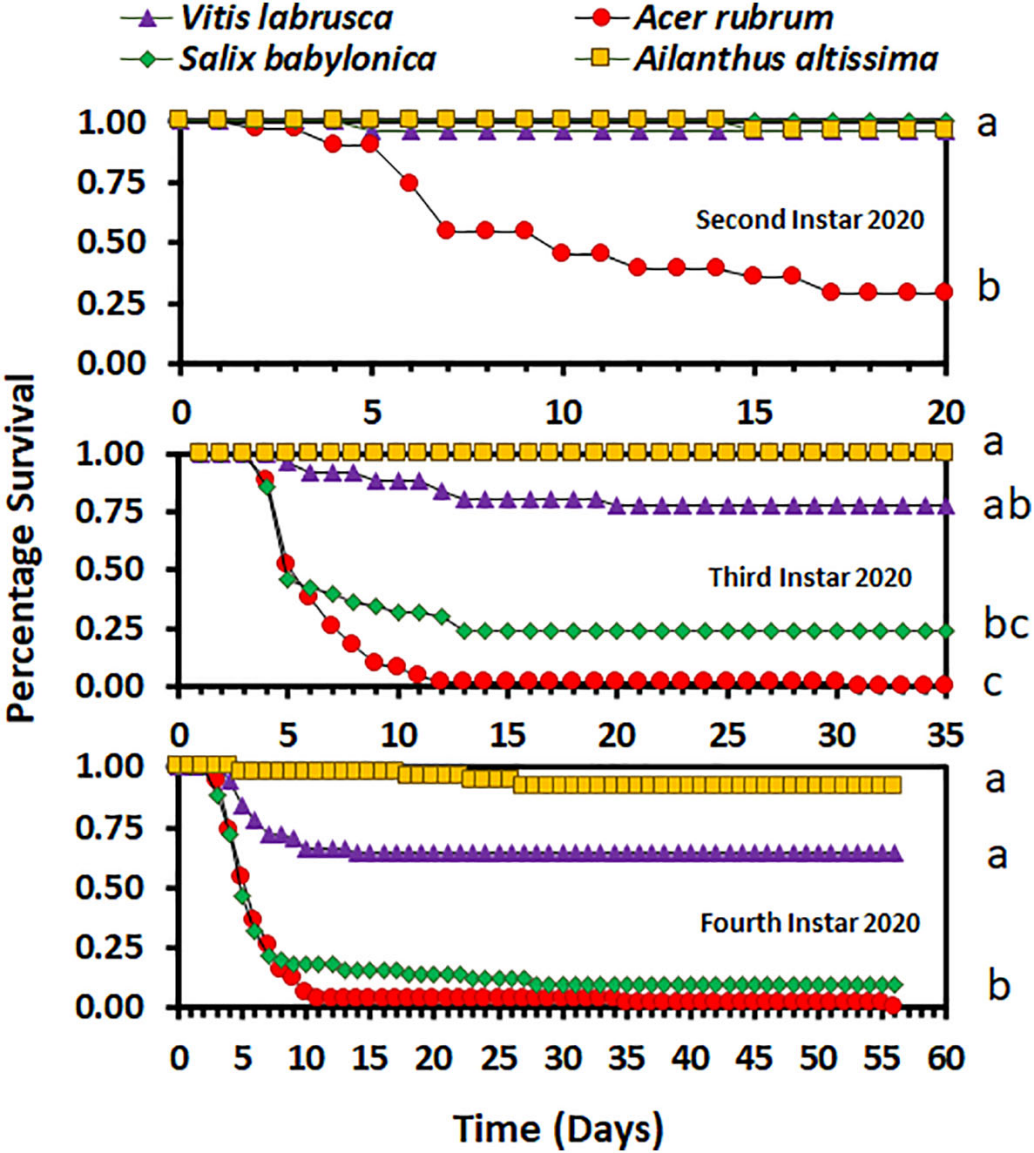
Survivorship curves for *Lycorma delicatula* nymphs reared in 2020 by instar and host plant species. Different letters to the right of the graphs indicate differences between the overall survival for the host plant species.

**Figure 2:**
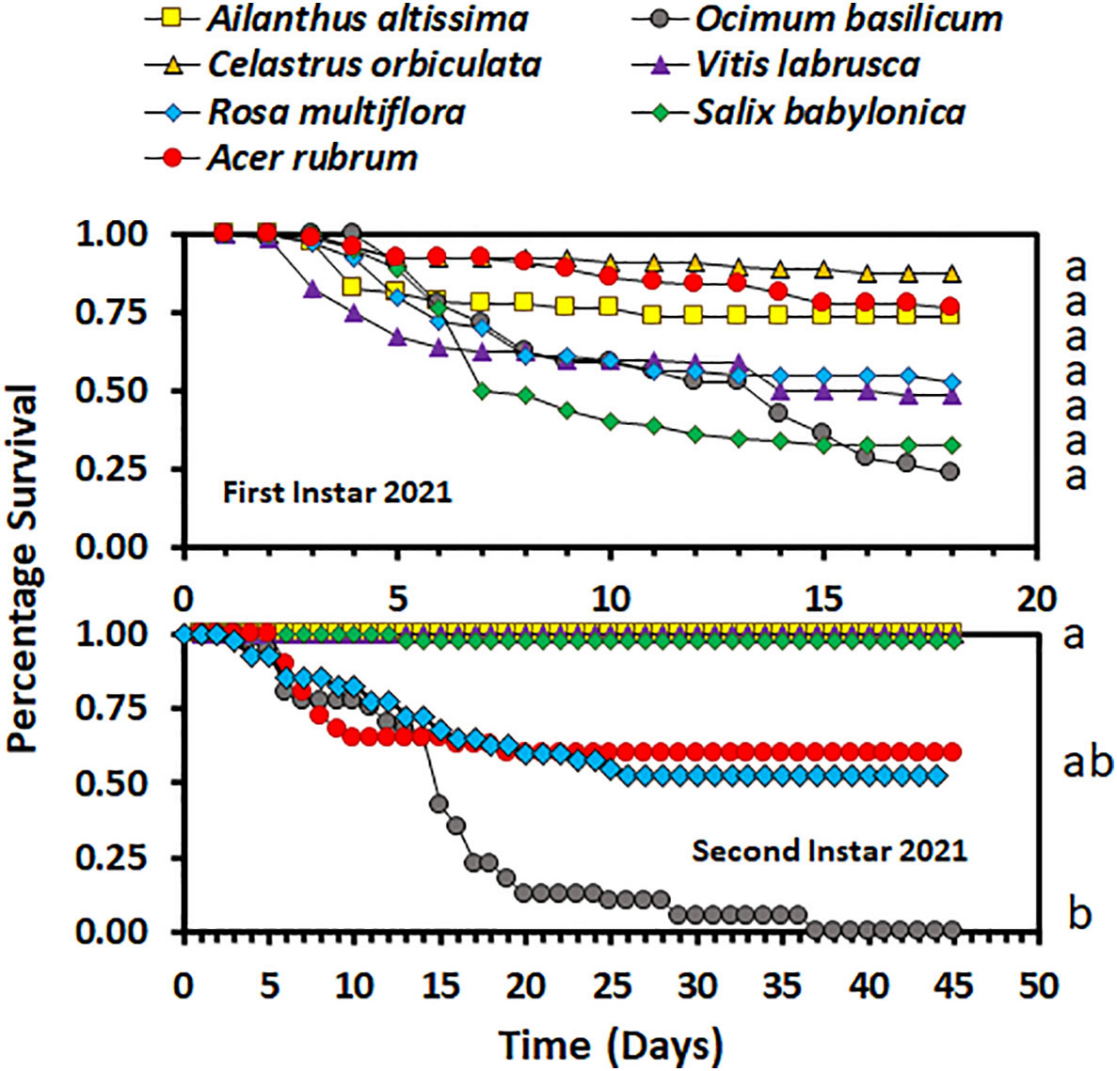
Survivorship curves for *Lycorma delicatula* nymphs reared in 2021 by instar and host plant species. Different letters to the right of the graphs indicate differences between the overall survival for the host plant species.

Overall, host plant species had an impact on nymphal survival. There was no significant difference in the survival curves (χ^2^ = 4.98, d.f. = 3, *p* = 0.1730), but there was a significant difference in overall survival (*F_3_*_,9_ = 5.03; *p* = 0.0256), between host plant species for the second-instar nymphs in 2020. In 2020, the overall percentage survival of second-instar nymphs was lowest on *A. rubrum* among all host plant species (**Figure 1**). The survival curves (χ^2^ = 114.44, d.f. = 3, *p* < 0.0001 for third-instar nymphs and χ^2^ = 100.48, d.f. = 3, *p* < 0.0001 for fourth-instar nymphs) and overall survival percentages (*F*_3,16_ = 5.12; *p* = 0.0113 for third instar nymphs, and *F*_3,36_ = 9.48; *p* < 0.0001 for fourth-instar nymphs) were significantly different between host plant species for both third- and fourth-instar nymphs in 2020. Third- and fourth-instar nymphs reared on *A. rubrum* and *S. babylonica* had the numerically lowest percentage survival. There was a significant difference in survival between host plant species for both the first- and second-instar nymphs in 2021 (χ^2^ = 87.12, d.f. = 6, *p* < 0.0001, for first-instar nymphs and χ^2^ = 55.00, d.f. = 6, *p* < 0.0001, for second-instar nymphs). There was no significant difference in overall survival by host for first-instar nymphs (*F*_6,21_ = 1.66; *p* = 0.1811), but there was a significant difference for second-instar nymphs (*F*_6,21_ = 3.44; *p* = 0.0159). In 2021, overall survival was numerically highest for first-instar nymphs reared on *C. orbiculate*, *A. altissima*, and *A. rubrum*, and lowest for those reared on *O. basilicum* and *S. babylonica*, but there were substantial differences between the individual cages in percentage survival (**Figure 2**). Second-instar nymphs had the highest overall percentage survival when reared on *C. orbiculate, A. altissima, S. babylonica*, and *V. labrusca*, and the lowest overall percentage survival when reared on *O. basilicum*.

### Nymphal development

#### 2020 development

**Figure 3:**
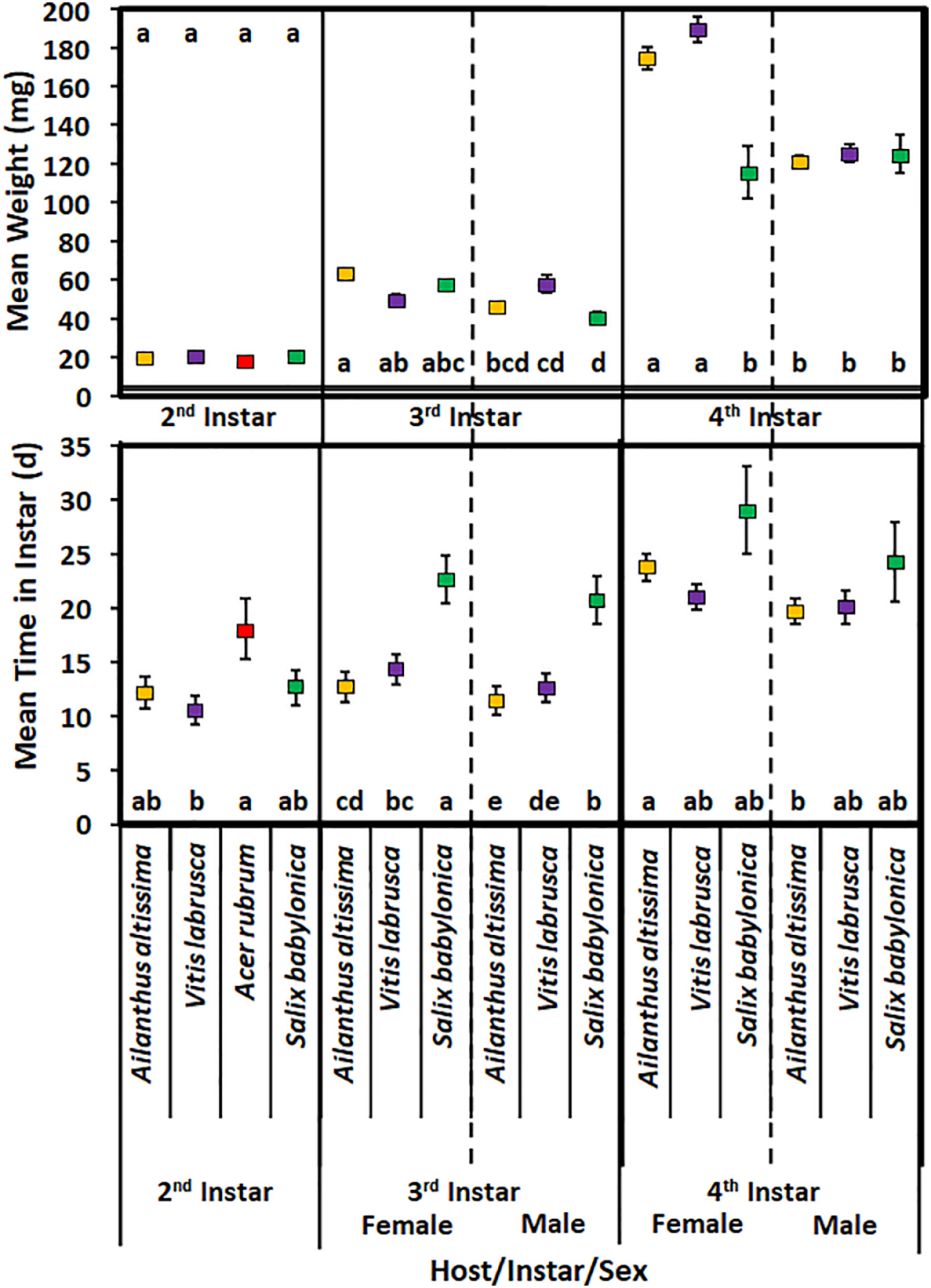
Mean time (days) spent in instar and weight (mg) of *Lycorma delicatula* nymphs reared in 2020 by host plant species, instar, and sex. Means with a different letter are significantly different from each other at a *p*-value < 0.05 using Tukey–Kramer grouping.

Host plant species did not have a significant effect on the mean time spent in the second instar in 2020 (*F*_3,78_ = 2.49; *p* = 0.0660) (**Figure 3**). Second-instar nymphs reared on *A. rubrum* spent significantly more time in that instar than nymphs reared on *V. labrusca*. In addition, host plant species did not have a significant effect on the weight of nymphs on completing the second instar (*F*_3,78_ = 0.91; *p* = 0.4400).

For third-instar nymphs, the mean development time was significantly affected by sex (*F*_1,186_ = 30.87; *p* > 0.0001), with female nymphs having longer development time (**Figure 3**). Host plant species had a significant effect on the mean development time in the third instar (*F*_2,186_ = 8.98; *p* = 0.0003); however, there was no significant difference for nymphs reared on *A. altissima* and *V. labrusca*. There was no significant interaction of host plant species and sex for third-instar nymphs (*F*_2,186_ = 0.35; *p* = 0.7029). Female nymphs reared on *S. babylonica* spent significantly longer in the third instar than all other nymphs. None of the nymphs reared on *A. rubrum* were able to complete the third instar.

Sex had a significant effect on the mean weight of nymphs that completed the third instar (*F*_1,186_ = 126.8; *p* < 0.0001) (**Figure 3**). For each host plant species, female nymphs weighed significantly more than male nymphs reared on the same host plant species. Neither host plant species (*F*_2,186_ = 1.68; *p* = 0.193) nor the interaction of host plant species and sex (F_2,186_ = 1.99; *p* = 0.143) had a significant effect on the mean weight of nymphs that completed the third instar. No significant difference was found in the mean weight of male or female nymphs reared on any of these hosts. In addition, female nymphs that were reared on *A. altissima* weighed significantly more than male nymphs that were reared on either S. *babylonica* or *V. labrusca*. Female nymphs reared on *V. labrusca* also weighed significantly more than male nymphs that were reared on *S. babylonica*.

Sex had a significant effect on the mean development time spent in the fourth instar (*F*_1,59_ = 7.26; *p* = 0.009) (**Figure 3**); however, host plant species did not have a significant effect on the mean time spent in the fourth instar (*F*_2,59_ = 0.88; *p* = 0.4195). Likewise, the interaction of host and sex also did not have a significant effect on the mean time spent in the fourth instar (*F*_2,59_ = 0.6; *p* = 0.5526). Female nymphs reared on *A. altissima* had a significantly longer developmental time than male nymphs that were reared on the same host plant. None of the fourth-instar nymphs reared on *A. rubrum* were able to complete the fourth instar.

#### 2021 development

**Figure 4:**
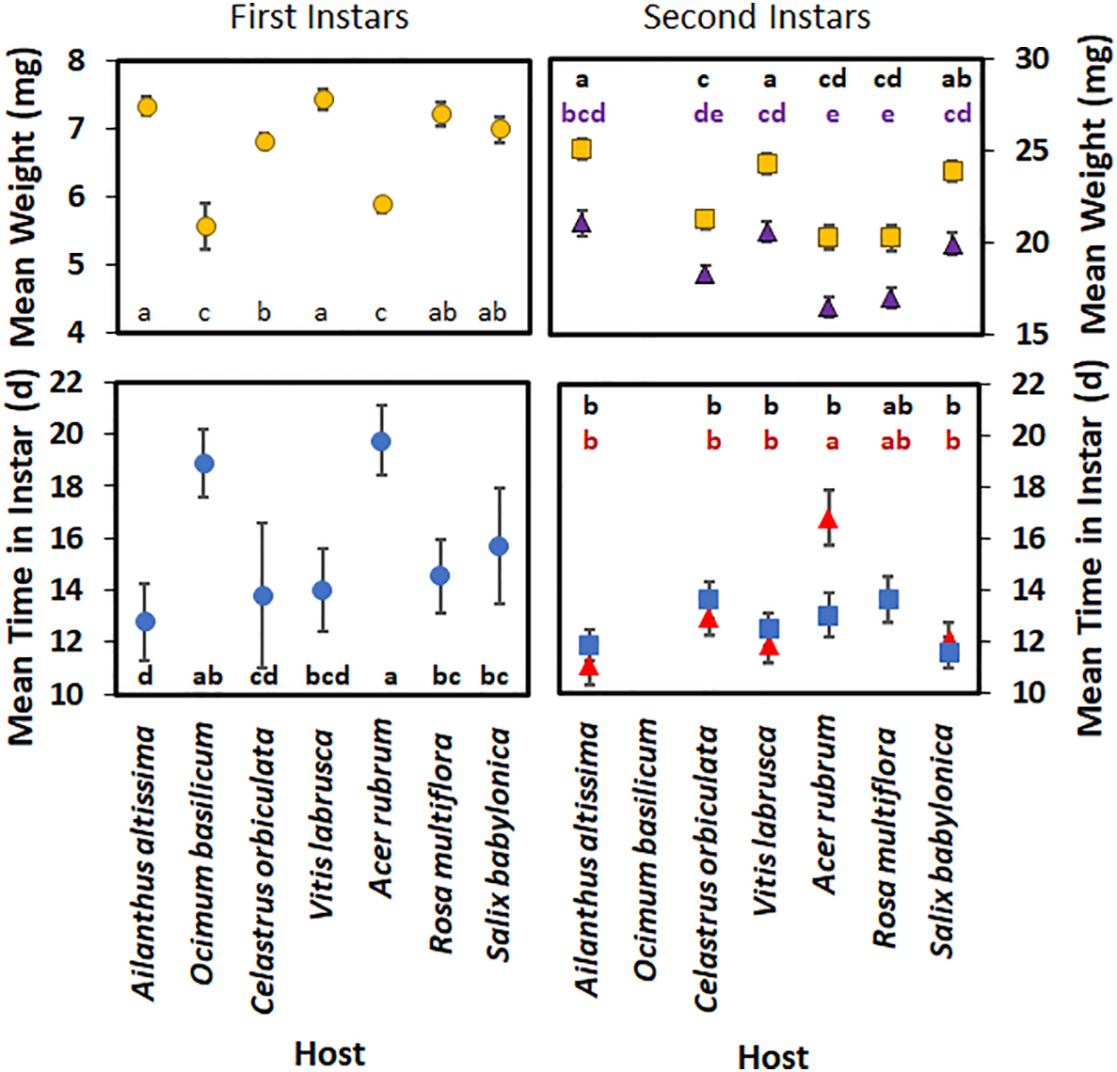
Mean time (days) spent in instar and weight (mg) of *Lycorma delicatula* nymphs reared in 2021 by host plant species and instar. Triangles represent males, whereas squares represent females for second instar nymphs. Means with a different letter are significantly different from each other at a *p*-value < 0.05 using Tukey–Kramer grouping.

For first-instar nymphs, host plant species had a significant effect on developmental time (*F*_6,262 = _24.21; *p* < 0.0001) (**Figure 4**), with nymphs reared on *A. rubrum* having a significantly longer developmental time than those reared on all other host plant species, except for *O. basilicum*. There was no significant difference in mean nymphal development time in first instar between those reared on *A. altissima*, *V. labrusca*, and *C. orbiculata*. First-instar nymphs also spent significantly less time in the first instar when reared on *A. altissima* than those reared on *R. multiflora*, *S. babylonica*, *A. rubrum*, and *O. basilicum*. The weights of first-instar nymphs were also significantly affected by the host plant species (*F*_6,271_ = 22.41; *p* > 0.0001), although no significant differences were observed in the weights of first-instar nymphs reared on *A. altissima*, *V. labrusca*, *R. multiflora*, and *S. babylonica*. Nymphs reared on *C. orbiculata* weighed significantly less than those reared on *V. labrusca* or *A. altissima*; however, no significant difference was seen when their weights were compared with those reared on *R. multiflora* or *S. babylonica*. Nymphs reared on *O. basilicum* and *A. rubrum* weighed significantly less than nymphs reared on all other hosts.

For second-instar nymphs, both host plant species (*F*_5,186_ = 9.25; *p* < 0.0001) (**Figure 4**) and the interaction of host plant species and sex (*F*_5,186_ = 3.25; *p* = 0.008) had significant effects on the mean development time in the second instar. Sex alone did not have a significant effect (*F*_1,186_ = 0.62; *p* = 0.432) on the mean development time in the second instar. When reared on *A. rubrum*, males spent significantly longer in that instar than females. In addition, male nymphs reared on *A. rubrum* took significantly longer than second-instar nymphs reared on any other host, except for *R. multiflora*. None of the nymphs reared on *O. basilicum* were able to complete the second instar.

For the second-instar nymphs, sex had a significant effect on their mean weight (*F*_1,186_ = 113.27; *p* < 0.0001) (**Figure 4**), with female nymphs weighing more than male nymphs. Host plant species also significantly impacted the mean weight of nymphs (*F*_1,186_ = 22.25; *p* < 0.0001); however, the interaction of host plant species and sex was not significant (*F*_1,186_ = 0.22; *p* = 0.956). Female nymphs reared on *S. babylonica* weighed significantly more than female nymphs reared on *C. orbiculate*; however, no significant difference was seen when their weights were compared with the weights of female nymphs reared on *V. labrusca*, *A. altissima*, and *S. babylonica*. Female nymphs reared on these hosts weighed significantly more than female nymphs reared on *C. orbiculata*, *A. rubrum*, or *R. multiflora*. No significant difference in weight was found between male nymphs reared on *V. labrusca*, *C. orbiculata*, *A. altissima*, and *S. babylonica*; however, male nymphs reared on either *V. labrusca*, *A. altissima*, or *S. babylonica* weighed more than male nymphs reared on either *R. multiflora* or *A. rubrum*.

### 2020 adult mass and morphometrics

Host plant species (*F*_2,59_ = 3.97; *p* = 0.024), sex (*F*_1,59_ = 32.42; *p* < 0.0001), and the interaction of host plant species and sex (*F*_2,59_ = 8.02; *p* = 0.001), had a significant effect on the mean weight of adults that completed the fourth instar in 2020 (**Table 2**). Adult female nymphs that completed the fourth instar, and which had been reared on either *A. altissima* or *V. labrusca*, weighed significantly more than male adult nymphs that completed the fourth instar, and which had been reared on *A. altissima*, *V. labrusca*, or *S. babylonica*, as well as female adult nymphs that completed the fourth instar and had been reared on *S. babylonica*. Likewise, there was no significant difference in the mean weight of male and female adult nymphs that completed the fourth instar and which had been reared on either *A. altissima* or *V. labrusca*. There was also no significant difference observed in the mean weight of adult male nymphs that completed the fourth instar and were reared on *A. altissima*, *V. labrusca*, and *S. babylonica*, or female adults that completed the fourth instar and were reared on *S. babylonica*.

**Table 2:** Mean [± SE (*n*)] adult *Lycorma delicatula* body weight (g), forewing length (mm) and width (mm), and hind tibia length (mm) at different combinations of host plant species and sex in *L. delicatula* reared on three host plants in 2020.

Measure	Host plant species and sex^a^						Statistics		
	*Ailanthus altissima*		*Vitis labrusca*		*Salix babylonica*				
	Male	Female	Male	Female	Male	Female	*F*	d.f.	*p*-value
Weight (g)	0.120 ± 0.003b (32)	0.175 ± 0.006a (14)	0.124 ± 0.004b (12)	0.187 ± 0.006a (20)	0.124 ± 0.01b (4)	0.115b (1)	8.02	2,59	0.001
Forewing length (mm)	17.43 ± 0.22b (31)	21.43 ± 0.39a (13)	18.01 ± 0.35b (12)	21.55 ± 0.33a (20)	17.65 ± 0.59b (4)	18.39ab (1)	2.42	2,68.73	0.097
Forewing width (mm)	7.683 ± 0.11b (31)	9.18 ± 0.19a (13)	7.852 ± 0.18b (12)	9.361 ± 0.11a (20)	7.585 ± 0.30b (4)	7.56ab (1)	2.01	2,75	0.142
Hind tibia length (mm)	9.875 ± 0.10b (31)	11.05 ± 0.16a (13)	10.08 ± 0.15b (12)	11.15 ± 0.14a (20)	9.79 ± 0.28b (4)	9.58ab (1)	2.84	2,69.31	0.066

a Means followed by the same letter are not significantly different at a p-value ≤ 0.05 using Tukey–Kramer grouping. Sample size (N) is the number of survivors.

d.f., degrees of freedom.

Sex had a significant effect on adult forewing length (*F*_1,64.92_ = 28.16; *p* < 0.0001), whereas host plant species (*F*_2,20.14_ = 2.81; *p* = 0.084) and the interaction of host plant species and sex (*F*_2,68.73_ = 2.42, *p* = 0.097) did not (**Table 2**). Both host plant species (*F*_2,75_ = 3.71; *p* = 0.029) and sex (*F*_1,75_ = 13.32; *p* = 0.001) had a significant effect on the forewing width of adult nymphs; however, the interaction of host plant species and sex (*F*_2,75_ = 2.01; *p* = 0.142) did not. Female adult nymphs reared on either *A. altissima* or *V. labrusca* had significantly wider and longer forewings than male nymphs reared on these hosts. Likewise, host plant species (*F*_2,19.75_ = 3.93; *p* = 0.037) and sex (*F*_1,64.45_ = 10.49; *p* = 0.002) had a significant effect on adult hind tibia length, whereas the interaction of host plant species and sex (*F*_2 69.31_ = 2.84; *p* = 0.066) had no significant effect. The hind tibia length of female adult nymphs that completed development on either *A. altissima* or *V. labrusca* was significantly longer than the hind tibia length of adult male nymphs reared on these two hosts.

## Discussion

Host plant species had an effect on nymphal survival. Nymphs reared on *A. altissima* and *V. labrusca* survived equally well, but survival was decreased for those reared on *R. multiflora*, *A. rubrum*, and *O. basilicum*. Nymphs failed to develop through the second instar on *O. basilicum* and through the third and fourth instars on *A. rubrum*. Host plant species was found to have a significant effect on the development time of *L. delicatula* nymphs in the first through third instars. Host plant species was also found to have a significant effect on the mean weight of nymphs in the first, second, and fourth, but not in the third, instars. Host plant species had a significant effect on adult hind tibia length and forewing width. These findings should be incorporated into phenology models for *L. delicatula* to account for host effects.

First-instar nymphs reared on *A. rubrum* and *O. basilicum* took longer to develop and later, as second-instar nymphs, had the lowest weights. The inability of second-instar nymphs reared on *O. basilicum* to complete the second instar, and of third- and fourth-instar nymphs reared on *A. rubrum* to complete development, suggests that the performance of earlier instars is indicative of host viability for later instars. Declining host viability as nymphal development progresses was also seen in the percentage survival of second- through fourth-instar nymphs in 2020; specifically, second-instar nymphs reared on *S. babylonica* had a similar percentage survival to those reared on either *A. altissima* or *V. labrusca*. This trend of reduced viability as development progresses can also be seen in previous research, in which a shift away from *R. multiflora* as the dominant host was observed in the *L. delicatula* third instar (13). In addition, this trend of reduced survival on hosts where nymphs take longer to develop in earlier instars is also seen in *H. halys* nymphs (8). Another study found that the number of host plants on which *L. delicatula* nymphs could complete the first instar was higher than the number on which it could complete the second instar (14). In addition, in that study, second-instar nymphs reared on *A. rubrum* failed to complete that instar, and third-instar nymphs reared on *S. babylonica* failed to complete that instar, supporting the results seen for those hosts with later-instar nymphs in this study. Interestingly, *L. delicatula* has consistently good survival during all instars on its preferred host*, A. altissima*. Furthermore, life stages where host plant species had a significant effect on the mean time spent in each instar, *A. altissima* was one of the hosts that nymphs spent the least amount of time feeding on during each instar, as inferred from the slow-growth, high-mortality hypothesis (15). In many cases, there was no significant difference in mean development time in instar between nymphs reared on *V. labrusca* and those reared on *A. altissima*, thus suggesting that *V. labrusca* is comparable to *A. altissima* as a host for *L. delicatula* nymphs. The interaction of host and sex had a significant effect on the mean time spent in instar only for second-instar nymphs; however, that is most likely a result of the fact that male second-instar nymphs reared on *A. rubrum* spent significantly longer in that instar than nymphs reared on other hosts, excluding *R. multiflora*.

Host plant species also had a significant effect on the mean weight of *L. delicatula* nymphs in the first and second instars. This difference in weight is more likely explained by nutritional differences in the host plant, rather than by differences in plant defensive compounds, as *L. delicatula* is known to sequester defensive compounds (16). Mean weight was also significantly affected by sex in the second, third, and fourth instars. The significant effect on mean weight of the interaction of host and sex in fourth-instar nymphs may limit the use of weight for sexing *L. delicatula* nymphs. The differences between sexes in development time and weight were reflective of each other, as the lighter males took less time to develop than the heavier females. Lower weights in fourth-instar nymphs were also associated with less optimal temperatures in previous research, which further hints at *S. babylonica* being a less optimal host than either *A. altissima* or *V. labrusca* for female fourth-instar nymphs (9). The general similarities in the mean time spent in an instar for first- and second-instar nymphs among different host plant species suggest that weight might be a better indicator of host suitability for those instars. For first-instar nymphs, longer developmental times also resulted in nymphs with lower weights. Growth rate affects the size of an individual, but the final size is determined by factors that terminate growth and lead to a molt. Many insects have a critical weight they must achieve before they molt and, if this weight is not reached, they do not survive. The critical weight has been determined for *Manduca sexta* L. (Lepidoptera: Sphingidae), and molting frequency is associated with growth rate (17). In addition, slower growth rate has been seen in *M. sexta* in response to suboptimal temperatures or nutrition, which matches the trend shown in this study for *L. delicatula*. Thus, it may be the case that, on suboptimal hosts, reach the critical weights for each instar only just before molting.

Adult morphometrics differed by sex, further suggesting that there is size-based sexual dimorphism in *L. delicatula* adults. Host plant species had a significant effect on the hind tibia length and forewing width of *L. delicatula* adults. These factors are more indicative of nymph size than forewing length, which has been shown to affect the flight capabilities of *L. delicatula* (18). In *L. delicatula* adults, weight appears to be a proxy for sex and nourishment level. Nourishment level could have an impact on nymph flight capabilities, and this is particularly important in the context of dispersal, as extra nourishment could be used to sustain longer flights (19). Heavier weights can also allow adult to persist longer without food sources, as seen with other hemipterans, and thus increase the odds of individual nymphs surviving human-mediated dispersal events, such as those occurring on planes or cargo ships (20). Landscape-level decisions, in terms of host quality for *L. delicatula*, could also play a role in dispersal through shipping hubs, ports, and airfields.

The results of this study have implications for phenology models for *L. delicatula* because phenology is affected by the host plant that individual nymphs feed on. Dynamic models accounting for host preference by instar are needed moving forward, so that accurate predictions of phenology can be made. As the mean development time did not differ significantly between nymphs reared on either *A. altissima* or *V. labrusca*, the degree-day requirements from Kreitman et al. (9) should be viable for degree-day modeling for monitoring the growth of *L. delicatula* in vineyards, where the development of *L. delicatula* on grape plants takes 12.6–12.77 days to complete. Regardless of which host the second-instar nymphs were reared on, the time spent in that instar was shorter than the time spent in the second instar at 25°C in the previously mentioned study. However, as the previous study did not account for sex in that instar, it could potentially not be a true comparison. Furthermore, the use of plastic tubes in that study and the use of the BugDorm cages in this one makes it harder to make comparisons because the cages could hold more, and larger, host plants. This same trend was also observed for nearly every host plant in the case of third-instar nymphs, with the exception of female third-instar nymphs reared on *S. babylonica*. The same trend was apparent with fourth-instar nymphs, which in both studies accounted for their sex. This difference in developmental rates between the two studies only confirms the disadvantages of using plastic tubes over other containers for rearing nymphs. This is different from previous research that found that both first- and second-instar nymphs took longer to develop on *Vitis rotundifolia* var. Carlos (Michaux) than on *A. altissima* (21). This suggests that *L. delicatula* nymphs perform differently depending on the variant and species of *Vitis* that they are reared on. Further studies that look at different host plant species and use a combination of host plant species similar to that found in forest and landscape environments are needed to get a better idea of how different host plant species influence the development of *L. delicatula* nymphs.

Overall, this study shows that the development of *L. delicatula* can be influenced by host plant species. Moving forward, it is important to consider potential host options when developing management strategies for *L. delicatula*. Furthermore, this research can be extrapolated to identify what nutrients *L. delicatula* require to complete development based on their host utilization. The differences in development time by host plant species indicate a potential issue regarding the use of phenology models to predict the current life stage. Sampling for field data to use for validating phenology models is important and might be affected at a site level by the host plants that are present. As being reared on certain host plants results in nymphs having a lower weight, this study can also inform host plant choice when mass rearing *L. delicatula* for potential parasitoid use. The risk of *L. delicatula* damage to *O. basilicum* in homeowner gardens seems to be minimal, as *O. basilicum* does not appear to be a viable host for later instar nymphs. It is important for further research evaluating *L. delicatula* nymphal utilization of other species of *Vitis* to be undertaken, as its presence is a major threat to grape production.

## Data availability statement

The raw data supporting the conclusions of this article will be made available by the authors, without undue reservation.

## Author contributions

Part of DK’s M.Sc. thesis. Authors jointly conceived the study and got the funding for it. MK and DK collected and analyzed the data. MK prepared the figures. DK wrote the first draft of the paper, and all authors edited the manuscript. All authors contributed to the article and approved the submitted version.

## References

[1] Barringer LE Donovall LR Spichiger SE Lynch D Henry D. The first new world record of *Lycorma delicatula* (Insecta: Hemiptera: Fulgoridae). Entomol News (2015) 125(1):20–3. doi: 10.3157/021.125.0105

[2] Barringer L Ciafre CM. Worldwide feeding host plants of spotted lanternfly, with significant additions from north America. Environ Entomol (2020) 49(5):999–1011. doi: 10.1093/ee/nvaa093 32797186

[3] Dara SK Barringer L Arthurs SP. *Lycorma delicatula* (Hemiptera: Fulgoridae): A new invasive pest in the united states. J Integr Pest Manage (2015) 6(1):20. doi: 10.1093/jipm/pmv021

[4] Uyi O Keller JA Johnson A Long D Walsh B Hoover K. Spotted lanternfly (Hemiptera: Fulgoridae) can complete development and reproduce without access to the preferred host, ailanthus altissima. Environ Entomol (2020) 49(5):1185–90. doi: 10.1093/ee/nvaa083 32725170

[5] Abarca M Larsen EA Lill JT Weiss M Lind E Ries L. Inclusion of host quality data improves predictions of herbivore phenology. Entomol Exp Appl (2018) 166(8):648–60. doi: 10.1111/eea.12715

[6] Awmack CS Leather SR. Host plant quality and fecundity in herbivorous insects. Annu Rev Entomol (2002) 47:817–44. doi: 10.1146/annurev.ento.47.091201.145300 11729092

[7] Myers CT Hull LA Krawczyk G. Effects of orchard host plants (apple and peach) on development of oriental fruit moth (Lepidoptera: Tortricidae). J Econ Entomol (2007) 100(2):421–30. doi: 10.1093/jee/100.2.421 17461067

[8] Acebes-Doria AL Leskey TC Bergh JC. Host plant effects on halyomorpha halys (Hemiptera: Pentatomidae) nymphal development and survivorship. Environ Entomol (2016) 45(3):663–70. doi: 10.1093/ee/nvw018 27012749

[9] Kreitman D Keena MA Nielsen AL Hamilton G. Effects of temperature on development and survival of nymphal lycorma delicatula (Hemiptera: Fulgoridae). Environ Entomol (2021) 50(1):183–91. doi: 10.1093/ee/nvaa155 33269378

[10] Park JD Kim MY Lee SG Shin CH Kim J Park IK. Biological characteristics of *Lycorma delicatula* and the conrtol effects of some insecticides. Korean J Appl Entomol (2009) 48(1):53–7. doi: 10.5656/KSAE.2009.48.1.053

[11] SAS Institute. SAS/STAT user's guide, version 9.4. Cary, NC: SAS Institute (2015).

[12] Analytical Software. Statistix 10 user's Manuel. Tallahassee, FL: Analytical Software (2013).

[13] Liu HP. Oviposition substrate selection, egg mass characteristics, host preference, and life history of the spotted lanternfly (Hemiptera: Fulgoridae) in north America. Environ Entomol (2019) 48(6):1452–68. doi: 10.1093/ee/nvz123 31651025

[14] Murman K Setliff GP Pugh CV Toolan MJ Canlas I Cannon S. Distribution, survival, and development of spotted lanternfly on host plants found in north America. Environ Entomol (2020) 49(6):1270–81. doi: 10.1093/ee/nvaa126 33128562

[15] Benrey B Denno RF. The slow growth high mortality hypothesis: a test using the cabbage butterfly. Ecology (1997) 78(4):987–99. doi: 10.1890/0012-9658(1997)078[0987:TSGHMH]2.0.CO;2

[16] Song S Kim S Won Kwon S Lee S-I Jabłoński P. Defense sequestration associated with narrowing of diet and ontogenetic change to aposematic colours in the spotted lanternfly. Sci Rep (2018) 8:16831. doi: 10.1038/s41598-018-34946-y 30442911 PMC6237927

[17] Callier V Nijhout HF. Body size determination in insects: a review and synthesis of size- and brain-dependent and independent mechanisms. Biol Rev (2013) 88(4):944–54. doi: 10.1111/brv.12033 23521745

[18] Wolfin MS Myrick AJ Baker TC. Flight duration capabilities of dispersing adult spotted lanternflies. Lycorma delicatula. J Insect Behav (2020) 33(2-4):125–37. doi: 10.1007/s10905-020-09754-w

[19] Johnson CG. Physiological factors in insect migration by flight. Nature (1963) 198:423–7. doi: 10.1038/198423a0

[20] Gergs A Jager T. Body size-mediated starvation resistance in an insect predator. J Anim Ecol (2014) 83(4):758–68. doi: 10.1111/1365-2656.12195 24417336

[21] Nixon LJ Jones SK Tang L Urban J Felton K Leskey TC. Survivorship and development of the invasive lycorma delicatula (Hemiptera: Fulgoridae) on wild and cultivated temperate host plants. Environ Entomol (2022) 51(1):222–8. doi: 10.1093/ee/nvab137 34864970

